# Integration analysis based on fatty acid metabolism robustly predicts prognosis, dissecting immunity microenvironment and aiding immunotherapy for soft tissue sarcoma

**DOI:** 10.3389/fgene.2023.1161791

**Published:** 2023-03-30

**Authors:** Binfeng Liu, Shasha He, Chenbei Li, Chengyao Feng, Hua Wang, Haixia Zhang, Chao Tu, Zhihong Li

**Affiliations:** ^1^ Department of Orthopaedics, The Second Xiangya Hospital of Central South University, Changsha, Hunan, China; ^2^ Hunan Key Laboratory of Tumor Models and Individualized Medicine, The Second Xiangya Hospital of Central South University, Changsha, Hunan, China; ^3^ Department of Oncology, The Second Xiangya Hospital of Central South University, Changsha, Hunan, China

**Keywords:** soft tissue sarcoma, fatty acid metabolism, prognosis, Immune infiltration, immunotherapy

## Abstract

**Background:** Soft tissue sarcoma (STS) is a highly malignant tumor with a dismal prognosis. Presently, the dysregulation of fatty acid metabolism has received increasing attention in tumor research, but fewer reports are relevant to STS.

**Methods:** Based on fatty acid metabolism-related genes (FRGs), a novel risk score for STS was developed utilizing univariate analysis and least absolute shrinkage selection operator (LASSO) Cox regression analyses in the STS cohort, which were further validated using the external validation cohort from other databases. Furthermore, independent prognostic analysis, C-index, ROC curves, and nomogram were carried out to investigate the predictive performance of fatty acid-related risk scores. We also analysed the differences in enrichment pathways, the immune microenvironment, gene mutations, and immunotherapy response between the two distinct fatty acid score groups. Moreover, the real-time quantitative polymerase chain reaction (RT-qPCR) was used to further verify the expression of FRGs in STS.

**Results:** A total of 153 FRGs were retrieved in our study. Next, a novel fatty acid metabolism-related risk score (FAS) was constructed based on 18 FRGs. The predictive performance of FAS was also verified in external cohorts. In addition, the independent analysis, C-index, ROC curve, and nomograph also revealed that FAS could serve as an independent prognostic factor for the STS patients. Meanwhile, our results demonstrated that the STS cohort in two distinct FAS groups had different copy number variations, immune cell infiltration, and immunotherapy responses. Finally, the *in vitro* validation results demonstrated that several FRGs included in the FAS exhibited abnormal expression in STS.

**Conclusion:** Altogether, our work comprehensively and systematically clarifies fatty acid metabolism’s potential roles and clinical significance in STS. The novel individualized score based on fatty acid metabolism may be provided as a potential marker and treatment strategy in STS.

## Introduction

Soft tissue sarcoma (STS) represents a very heterogeneous group of malignant soft tissue neoplasms, accounting for 1% of all malignancies, with case fatality rates as high as 50% (Schuetze, 2005). According to the French Federation Nationale des Centres de Lutte Contre le Cancer (FNCLCC) grading system, STS can be divided into Ⅰ, Ⅱ, and Ⅲ grades (Coindre, 2006). Currently, STS is mainly treated by surgical resection in combination with adjuvant radiotherapy and chemotherapy. Nevertheless, the effectiveness is far from satisfactory for STS patients with various histological subtypes of STS and the existence of drug resistance (Liu et al., 2021). With substantial progress in genomics, transcriptomics, proteomics, epigenetics, and immunology research in recent years, targeted therapy and immunotherapy are becoming potential options for tumor treatment, but they are still in their infancy compared with traditional therapy (Liu et al., 2022a; Zhang et al., 2022). Therefore, identifying and characterizing novel biomarkers and implementing individual precise therapy is crucial for improving STS quality of life and prognosis.

Fatty acid metabolism is a crucial cellular procedure that disciples nutrients and energy into biosynthetic metabolic intermediates for membrane biosynthesis, energy storage, and signaling molecule generation (Currie et al., 2013; Röhrig and Schulze, 2016). Notably, the dysregulation of lipid synthesis and fatty acid oxidation genes was proven to be correlated with malignant phenotypes, including metastasis, recurrence, and resistance (Currie et al., 2013). For instance, fatty acid synthase (FASN) could regulate the injuries induced by genotoxicity by promoting PARP-1 and DNA repair *via* NF-κB and SP1 (Wu et al., 2016). In addition, it was recently demonstrated that fatty acid metabolism regulates the immune microenvironment, including T-cells and macrophages (Lochner et al., 2015; Van den Bossche et al., 2017; Raud et al., 2018). Also, accumulating research has demonstrated the role of subtypes based on fatty acid metabolism-related genes (FRGs) in numerous tumors. As an example, Ding et al. demonstrated that a novel signature based on fatty acid metabolism-related gene cloud serves as a predictor for the prognosis, chemotherapy, and immunotherapy response for colorectal cancer (Ding et al., 2021). Hence, it is reasonable to believe that enhanced knowledge of the relevance between fatty acid metabolism and STS may be expected to provide novel insight into tumor progression and immunotherapy.

With rapidly advancing sequencing technology, various scale sequencing data are becoming increasingly accessible and could effectively help researchers comprehensively reveal the molecular characteristics of tumors. Herein, we systematically explored the association of STS with FRGs and developed and verified a novel fatty acid metabolism score (FAS). Next, we determined the prediction value of FAS using K-M survival analysis, C-index, ROC curve, external validation, and independent analysis. Moreover, we also comprehensively analysed the differences in somatic mutations, immunity microenvironment, and immunotherapy response between high- and low-FAS patients using a series of bioinformatic methods. Hence, this study will be helpful for early prognosis prediction and individualized guide treatment for STS.

## Methods and materials

### Data source

RNA sequencing, mutation data, HumanMethylation450 array data, and corresponding clinical features were downloaded from The Cancer Genome Atlas (TCGA, https://www.cancer.gov/aboutnci/organization/ccg/research/str uctural-genomics/tcga) database. A total of 492 STS individuals were enrolled after removing the patient’s unfollowed and incomplete or erroneous clinical characteristics. Meanwhile, the STS cohort from The Therapeutically Applicable Research to Generate Effective Treatments database (TARGET, https://ocg.cancer.gov/programs/target) was extracted from the UCSC Xena Hub datasets. GSE21050 and GSE71118 obtained from the UCSC Xena Hub datasets ( https://xena.ucsc.edu/) the Gene Expression Omnibus (GEO, https://www.ncbi.nlm.nih.gov/geo/). were external validation cohorts to verify progression-free survival (PFS). The immunotherapy dataset included 298 individuals with metastatic urothelial tumors treated with anti-PD-L1 and was utilized to explore the immunotherapy response of STS.

### Generation and validation of FAS

The FRGs were obtained from hallmark gene sets in the Molecular Signatures Database (MSigDB, https://www.gsea-msigdb.org/gsea/msigdb/collections.jsp), and their detailed information is illustrated in Supplementary Table S1. To identify the abnormally expressed FRGs in STS, we extracted normal soft tissue from the Genotype-Tissue Expression (GTEx, https://www.gtexportal.org/home/) database, which served as a normal control. The threshold for screening differential genes was set as log2|fold change| > 1, FDR <0.05. Additionally, the selection of prognosis-related genes (*p* < 0.05) was performed by univariate Cox regression analysis. The intersection of differentially expressed FRGs and prognosis-related genes was used for further analysis. Subsequently, least absolute shrinkage and selection operator (LASSO) penalized Cox regression was performed to screen optimal prognostic features. To control overfitting, we implemented fivefold cross-validation and performed 300 iterations to determine the most robust and stable model. Finally, the genes with the highest frequency were used for signature construction, and the FAS was computed through the following formula: $FAS=\sum iCoefficient\left({mRNA}_{i}\right)\times Expression\left({mRNA}_{i}\right)$ .

As we all know, the larger C‐index indicates a more accurate prognostic prediction, so the R package “survcomp” was further utilized to acquire the concordance index (C-index) for assessment of the predictive prediction ability of FAS in each cohort. In addition, the distinct risk groups were classified by median FAS. Then, the K-M survival curve, Cox regression, and ROC curve were applied to assess the prognostic value of FAS.

### Function and immune infiltration analysis

To characterize signaling pathways associated with FAS, we first performed Gene Ontology (GO) analysis with Metascape (http://www.m etascape.org). Next, we performed gene set enrichment (GSEA) analysis between the distinct FAS groups and screened for important KEGG pathways (*p* < 0.05). Additionally, the “gsva” package was utilized to conduct a ssGSEA analysis based on molecular markers from previously published research for evaluating sample immune-related pathway activity. Detailed information on these molecular markers is provided in Supplementary Table S2. To investigate the relationships between FAS and immune cell infiltration, we not only estimated the infiltration abundance of immune cells in STS samples by the “CIBERSORT” package but also assessed the immune activity and tumor purity of the STS cohort *via* the Estimate algorithm. Ultimately, we collected the homologous recombination defects (HRD) score, intratumor heterogeneity, indel neoantigens, and SNV neoantigens of the individual from Thorsson et al. (Thorsson et al., 2018) to comprehensively explore the characteristics of the FAS.

### Genomic variant analysis

To investigate the difference in tumor mutation between the two distinct FAS groups, we applied “maftools” to process the mutation data. Initially, we calculated the total number of mutations in each sample and then screened the genes with a minimum number of mutations >8. Moreover, the differences in gene mutation frequencies between the high and low FAS groups were compared using the chi-square test and visualized with maftools. GISTIC software was utilized to process the CNV data and identify the significantly amplified or deleted genomic regions, and these results were visualized by the R package “GGplot2”. Finally, we further explored the mutational signatures using the R package “Sigminer”, which could help us extract, analyse and visualize signatures from genomic alteration records to provide new insights into cancer research.

### Evaluation of immunotherapy response

Considering the importance of immunotherapy for tumors, we calculated the immunophenoscore (IPS) of each patient according to the totality of the weighted average Z score. IPS reflects the immune microenvironment of STS, and a higher IPS shows more robust responses to immunotherapy. Moreover, we employed Tumor Immune Dysfunction and Exclusion (TIDE) to predict the potential response to immune checkpoint blockade based on pretreatment tumor gene profiles that integrate the expression signatures of T-cell dysfunction and T-cell exclusion to model the mechanisms of tumor immune evasion. Ultimately, the efficacy of FAS for immunotherapy response prediction was verified in the immunotherapy cohort Imvigor210.

### Cell culture and RT-qPCR

The source and culture conditions of the STS cell lines (SW872 and SW982) and normal human skin fibroblast cell lines used in our study are listed in Supplementary Table S3. All cell lines were cultured at 37 °C in a humidified atmosphere with 5% carbon dioxide. RNA extraction, cDNA synthesis, and RT-qPCR reactions were performed according to the procedures described in previous studies (Liu et al., 2022a; Liu et al., 2022b). Meanwhile, GAPDH was selected as an internal reference, and the relative expression of FRGs was calculated through the 2^−ΔΔCT^ method. The primer sequences applied for RT-qPCR are presented in Supplementary Table S4.

### Statistical analysis

All statistical analyses and drawings in our study were carried out using R software. The differences between the two distinct FAS groups were compared by the Wilcoxon rank sum test for the comparison of medians and the chi-square test for the comparison of proportions. The Kaplan-Meier method was used to draw the survival curve, and we applied the log-rank test to examine the significance of differences. For time-dependent ROC curve, it was plotted by the “survivalROC” package. Besides, the R package “survival” was used to perform Cox regression analysis, and the “rms” was conducted to generate a nomogram and calibrated curve.

## Results

### The differentially expressed fatty acid-related gene in STS

**FIGURE 1:**
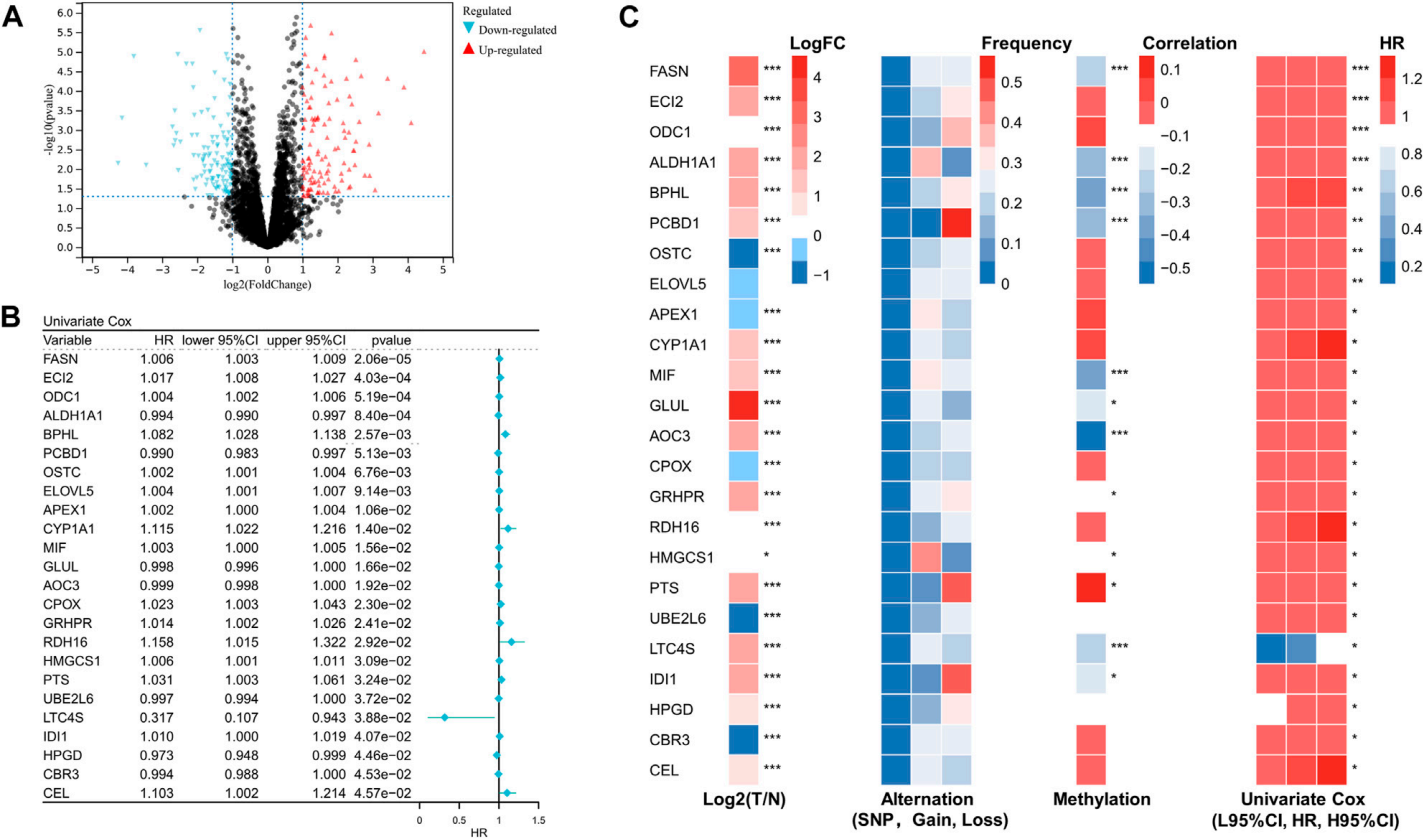
Identification of prognostic fatty acid metabolism related-gene in soft tissue sarcoma. **(A)** Volcano map of the differentially expressed genes associated with prognosis in the STS cohort. **(B)** Univariate analysis of differentially expressed fatty acid metabolism-related genes in STS. **(C)** The expression, alteration, and methylation level of differentially expressed fatty acid metabolism-related genes in STS.

Using differential gene expression (DEG) analysis, we discovered differentially expressed genes between STS and normal tissue. The differentially expressed genes associated with prognosis are shown in Figure 1A. After performing univariate Cox regression on these differentially expressed FRGs, we found that 24 differentially expressed FRGs could act as independent prognostic factors in STS. The forest plot of univariate Cox regression analysis was demonstrated in Figure 1B. As presented in Figure 1C, most of these 24 prognostic FRGs were dysregulated in STS. These findings prompted us to investigate somatic copy number alterations in these FRGs and reveal universal copy number changes in all 24 FRGs (Figure 1C). Among them, ALDH1A1 and MIF, with widespread copy number variation, were significantly elevated in STS. In contrast, ODC1 with copy number variation loss was expressed at a lower level in STS, hinting that copy number variation loss might regulate the expression of FRGs. DNA methylation, as one of the most common RNA modification, could regulate gene expression. We noted that several FRGs exist in methylation, such as PTS, CBR3, and CEL (Figure 1C). Collectively, these results revealed that these FRGs show a different genetic landscape and expression levels between STS and normal tissues, indicating their potential role in the oncogenesis of STS.

### The somatic mutation landscape of the FRGs

**FIGURE 2:**
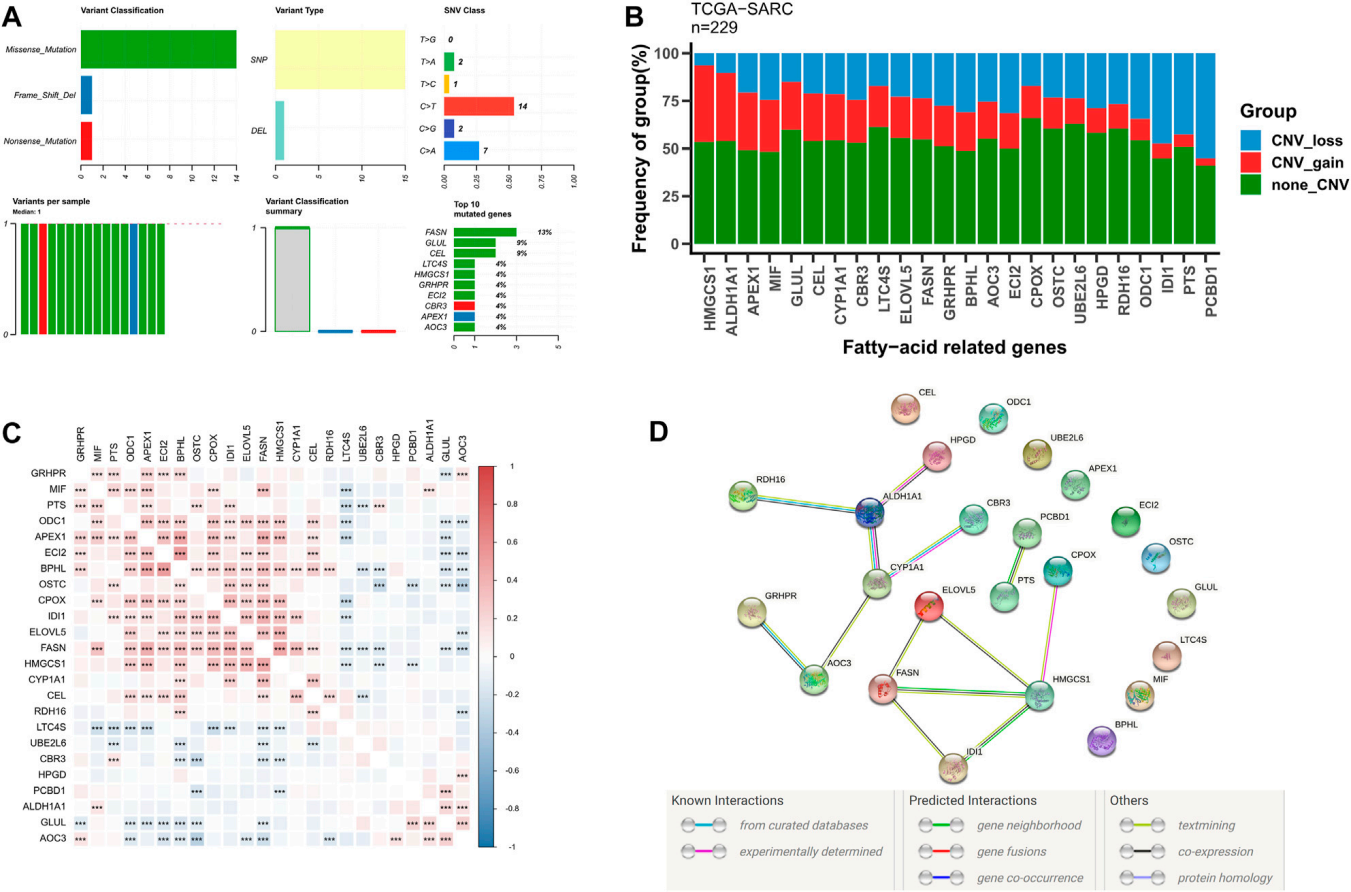
The genetic alterations and association of fatty acid metabolism-related genes in STS. **(A)** The somatic mutational status of 24 candidate fatty acid genes in STS. **(B)** The frequencies of CNV gain, loss, and non-CNV among fatty acid genes. **(C)** Heatmaps illustrating patterns of fatty acid gene coexpression. **(D)** PPI network map of fatty acid genes using STRING.

Subsequently, we further evaluated the somatic mutational status of these 24 candidate fatty acid genes. The results indicate that the main types of somatic mutations in the 24 candidate fatty acid genes are missense and SNP, of which FASN is the gene with the highest mutation frequency (Figure 2A). Simultaneously, most of these fatty acid-related genes have copy number variation, among which HNGCS1, ALDH1A1, and APEX1 have extensive CNV enhancement, while IDI1, PTS, and PCBD1 show CNV loss (Figure 2B). Additionally, we also discovered that these 24 fatty acid-related genes were significantly correlated with each other (Figure 2C), and their protein-protein interaction (PPI) network is also indicated in Figure 2D. These findings further confirm the potential role of these 24 candidate fatty acid genes in STS.

### Constructing and validating a novel fatty acid score

**FIGURE 3:**
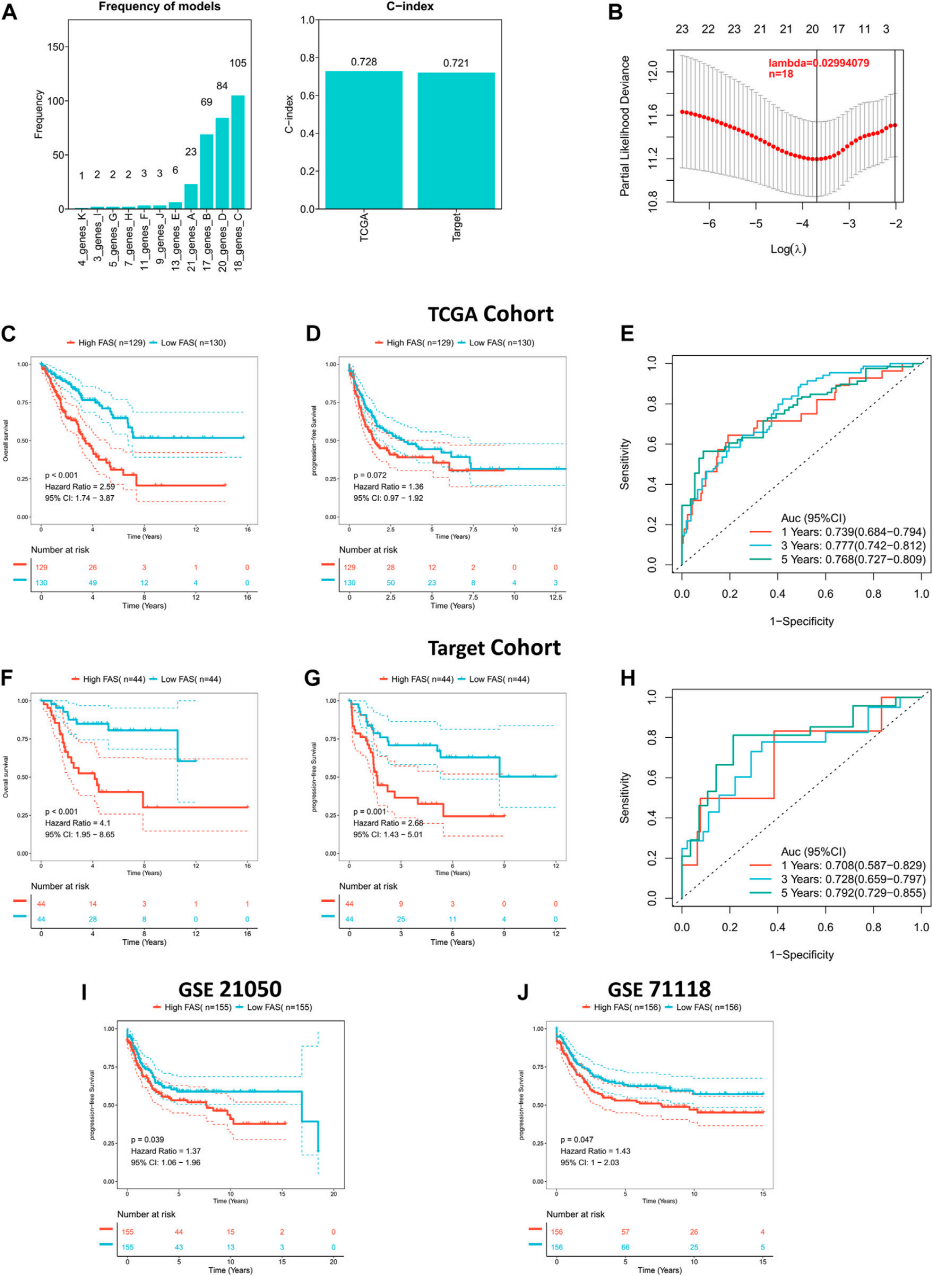
Construction of the fatty acid metabolism risk score (FAS) in STS. **(A)** The frequency and C-index of FAS consisted of 18 fatty acid metabolism-related genes. **(B)** LASSO coefficient profile analysis to identify the most helpful risk score system. **(C)** Kaplan‒Meier curves of OS in the TCGA cohort based on FAS. **(D)** Kaplan‒Meier curves of PFS in the TCGA cohort based on FAS. **(E)** ROC results of the FAS for the prediction of OS at 1, 3, and 5 years in the TCGA cohort. **(F)** Kaplan‒Meier curves of OS in the TARGET cohort based on FAS. **(G)** Kaplan‒Meier curves of PFS in the TARGET cohort based on FAS. **(H)** ROC results of the FAS for the prediction of OS at 1, 3, and 5 years in the TARGET cohort. **(I)** Kaplan‒Meier curves of PFS in GSE21050 based on FAS. **(J)** Kaplan‒Meier curves of PFS in GSE71118 based on FAS.

Considering the role of fatty acid-related genes in soft tissue sarcoma, we constructed an FAS based on the STS cohort from TCGA. As illustrated in Figures 3A, B, the fatty acid-related score constructed by 18 FRGs had the best prognostic value. The correlation coefficients of the 18 FRGs are shown in Supplementary Table S5. The C-index of FAS in the TCGA and TARGET cohorts was greater than 0.7, suggesting that the model’s prediction ability was accurate (Figure 3A). Subsequently, the survival curve presented that FAS was relevant to the survival prognosis of STS. With the increase in the fatty acid-related risk score, patients’ overall and progression-free survival decreased (Figures 3C, D). Meanwhile, the ROC curve indicated that the AUCs of the FAS at 1, 3, and 5 years were 0.739, 0.777, and 0.768, respectively (Figure 3E). Additionally, the external validation results based on the TARGET cohort indicated that the prognosis of patients with high FAS was poor (Figures 3F, G), and the AUC of the ROC curve was more significant than 0.7 (Figure 3H). Also, the external validation in GSE21050 and GSE71118 was consistent with the above results. The high FAS individuals had a shorter progression-free survival (Figures 3I, J). To sum up, this series of results revealed that the scoring system containing 18 fatty acid-related genes was successfully constructed and was closely associated with the clinical prognosis of STS.

### The prognosis and prediction value of FAS

**FIGURE 4:**
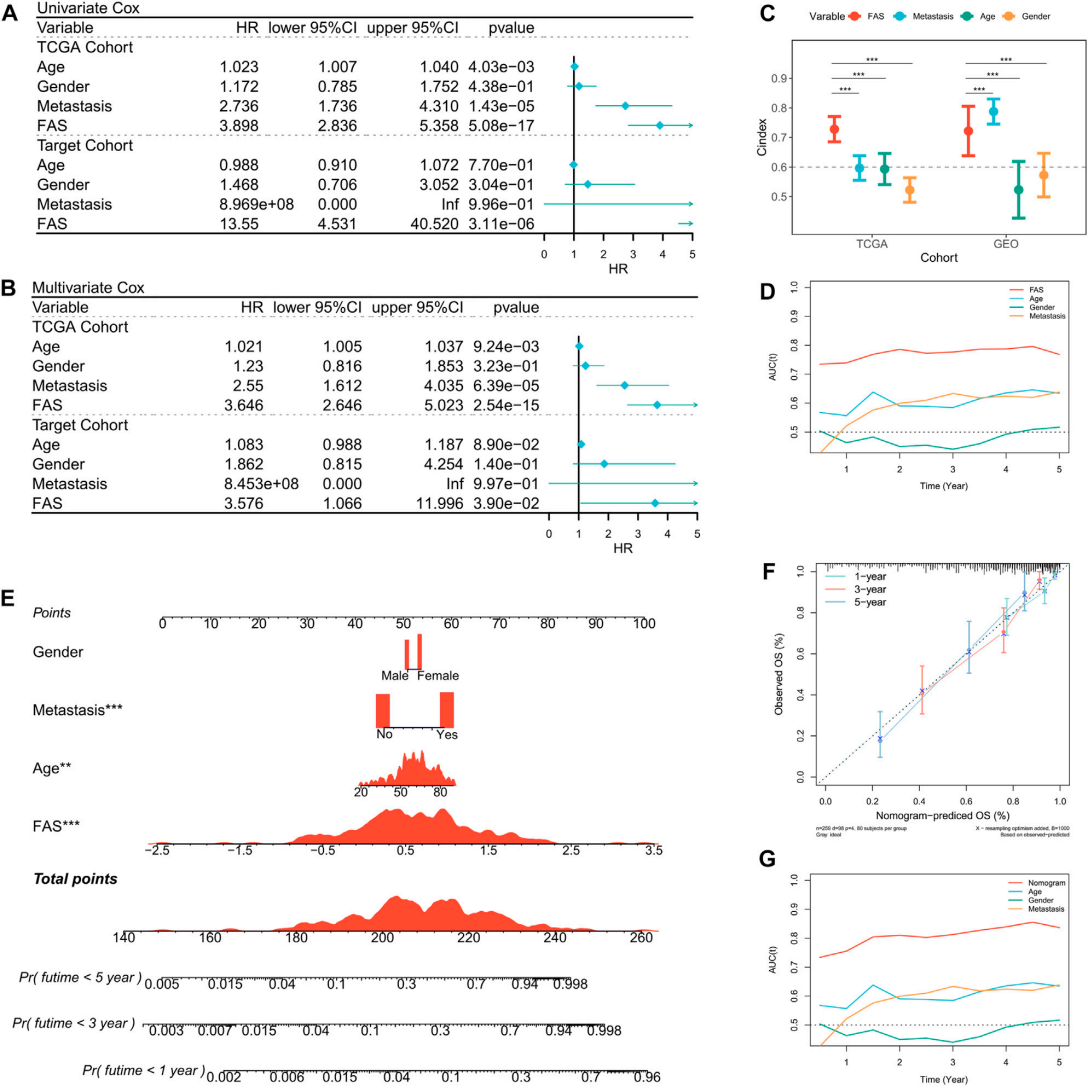
Prediction value of the FAS in STS. **(A)** The forest plot of the TCGA and TARGET cohort’s univariate Cox regression analysis. **(B)** The forest plot of the TCGA and TARGET cohort’s multivariate Cox regression analysis. **(C)** The C-index of the FAS in TCGA and GEO cohorts compared to other clinical characteristics. **(D)** The AUC of the FAS and clinical features. **(E)** The nomogram consists of FAS and other clinical characteristics to predict 1-, 3-, and 5-year OS in the TCGA-STS cohort. **(F)** The nomogram calibration plots. The y-axis represents actual survival, whereas the x-axis represents nomogram-predicted survival. **(G)** The AUC of the nomogram and clinical features.

Based on the prognosis prediction effect of FAS in STS, we further evaluated the predictive value of FAS. Univariate Cox regression analysis in the TCGA and TARGET cohorts showed that the fatty acid-related score was related to the prognosis of STS patients (Figure 4A). Multivariate COX regression analysis further demonstrated that the fatty acid-related score was an independent prognostic factor for patients with STS (Figure 4B). Additionally, compared with other clinical characteristics, the FAS had higher C-index and AUC values (Figures 4C, D), indicating that the model has superior prediction ability. In order to better predict the survival prognosis of patients with STS, we further established a nomograph based on FAS and clinical characteristics (Figure 4E). This nomograph can help us better predict the 1-year, 3-year, and 5-year survival rates of patients with STS. At the same time, the calibration curve showed that the predicted survival rate by nomogram is in good agreement with the actual survival rate (Figure 4F). The ROC curve also revealed that the AUC of the curve was significantly higher than that of other clinical characteristics (Figure 4G). These results confirmed that the scoring system composed of 18 fatty acid-related genes has robust value for the prognosis assessment of STS.

### The functional characteristics of FAS

**FIGURE 5:**
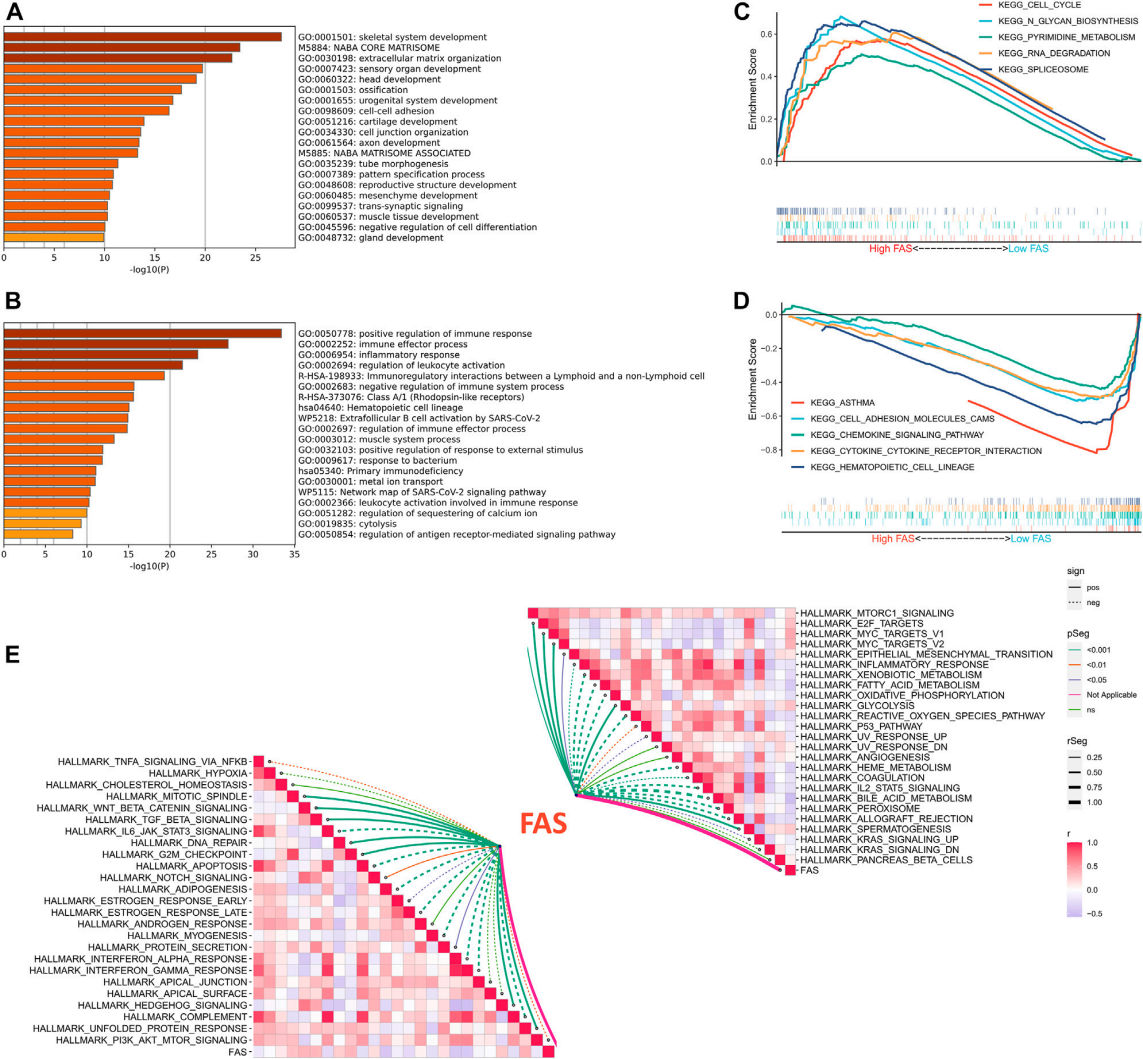
Functional analysis of FAS in STS. **(A,B)** GO enrichment analysis results for the low- and high-FAS groups in the TCGA-STS cohort. **(C)** The GSEA results of the high-FAS group in the TCGA-STS cohort. **(D)** GSEA results of the low-FAS group in the TCGA-STS cohort. **(E)** The association of FAS with molecular markers in the TCGA-STS cohort.

We further explored the biological function of FAS in STS, GO enrichment results implied that patients with a high fatty acid score in the TCGA and TARGET cohorts were significantly enriched in skeletal system development and extracellular matrix organization (Figure 5A; Supplementary Figure S1A), while positive regulation of the immune response, immune effector process, inflammatory response, and regulation of leukocyte activation was enriched in the low FAS cohort (Figure 5B; Supplementary Figure S1B). Meanwhile, we also conducted GSEA analysis, and the results revealed that Cell cycle, N-Glycan biosynthesis, Pyrimidine metabolism, RNA degradation, and splicesome were more active in the STS patients with high fatty acid scores (Figure 5C). On the other hand, patients with low FAS were more focused on immune-related functional pathways, such as the cell adhesion molecules cams, chemokine signaling pathway, cytokine-cytokine receptor interaction, natural killer cell-mediated cytotoxicity, and NOD-like receptor signaling pathway (Figure 5D; Supplementary Figure S1D). Since carcinogenesis is a complex process, we also explored the correlation between FAS and common signaling pathways. There was an inverse correlation between carcinogenesis-related pathways and FAS, such as WNT beta-catenin signaling and TGF beta signaling (Figure 5E; Supplementary Figure S1E). Meanwhile, FAS was negatively correlated with the P53 pathway and apoptosis (Figure 5E; Supplementary Figure S1E).

### Association between immune infiltration and FAS

**FIGURE 6:**
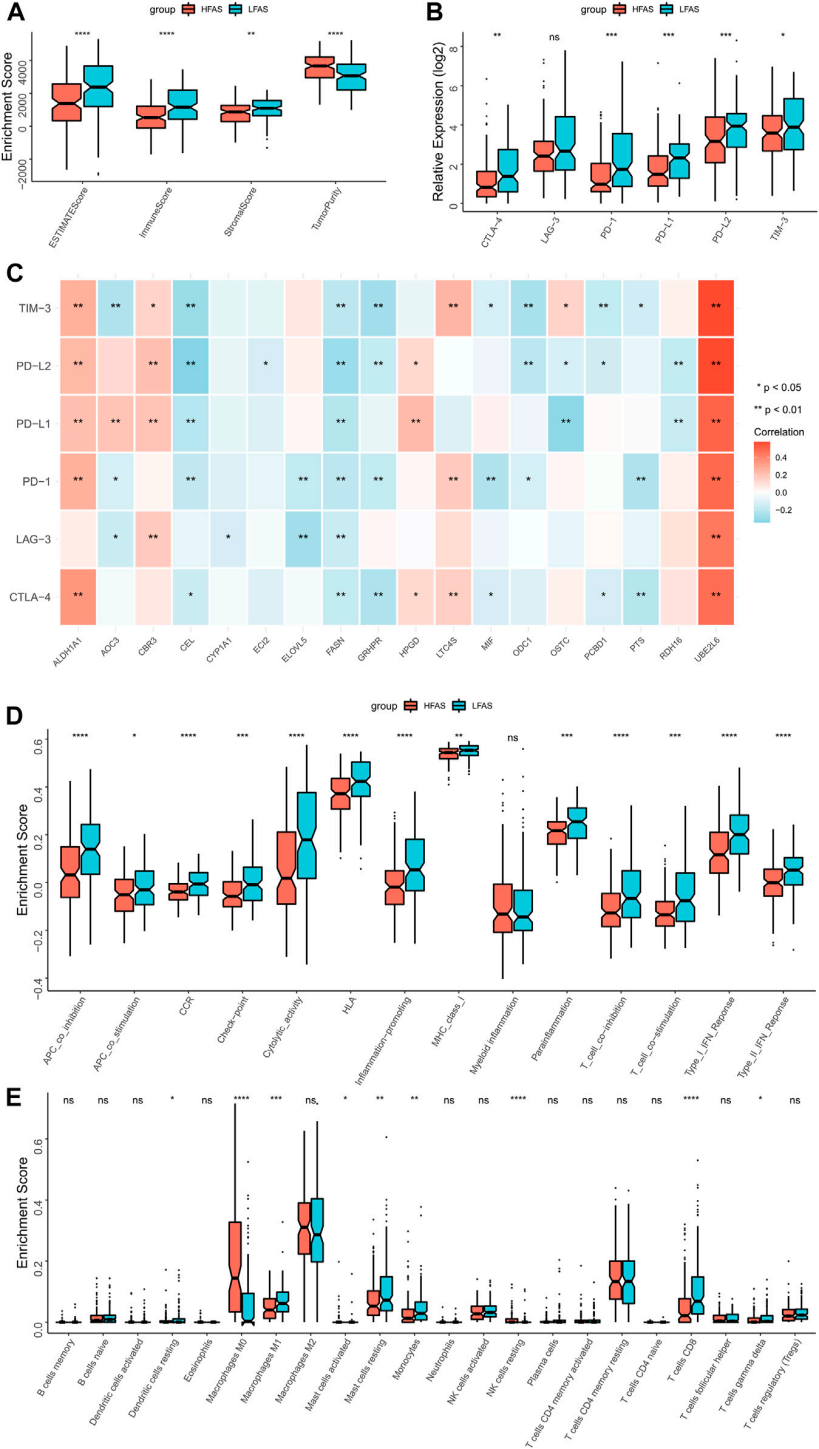
The immune landscapes between the high- and low-FAS groups in the TCGA-STS cohort. **(A)** The immune score between the low- and high-FAS groups. **(B)** The difference in immune checkpoint expression between the distinct FAS groups. **(C)** The relationship between the immune checkpoint and the FAS gene. **(D)** Analysis of immune function in high- and low-FAS patients. **(E)** The proportions of 22 immune-infiltrating cells between the distinct FAS groups.

Based on the correlation between the fatty acid correlation score and immune function, we further analysed the relationship between FAS and the immune microenvironment. The ESTIMATE results revealed that individuals with low FAS had higher immune scores, while tumor purity was lower in the STS or TARGET cohorts (Figure 6A; Supplementary Figure S2A). Next, we further analyzed the association between FAS and immune checkpoints and found that PD-L1, PD-L2, and TIM3 were more expressed in the low-FAS group (Figure 6B; Supplementary Figure S2B). At the same time, the expression of fatty acid-related genes ALDH1A1 and UBE2L6 was significantly correlated with the expression of immune checkpoints (Figure 6C; Supplementary Figure S2C). Later, we found that most of the immunity-related function pathways were brisker in STS patients with high FAS (Figures 6C, D; Supplementary Figure S2D) through ssGSEA analysis. As the immune cells played an important role in the immunity microenvironment, the association with the FAS was also investigated. The result indicated that the infiltration abundance of monocytes in the low-FAS group was higher than in the high-FAS group, both in the TCGA and TARGET cohorts (Figure 6E; Supplementary Figure S2E). Moreover, the low-FAS patients in the TCGA STS cohort also had a better infiltration level of Dendritic cells resting, Macrophages M1, Mast cell activated, Mast cell resting, and T cell CD8 (Figure 6E).

### The correlation between FAS and tumor mutation burden (TMB)

**FIGURE 7:**
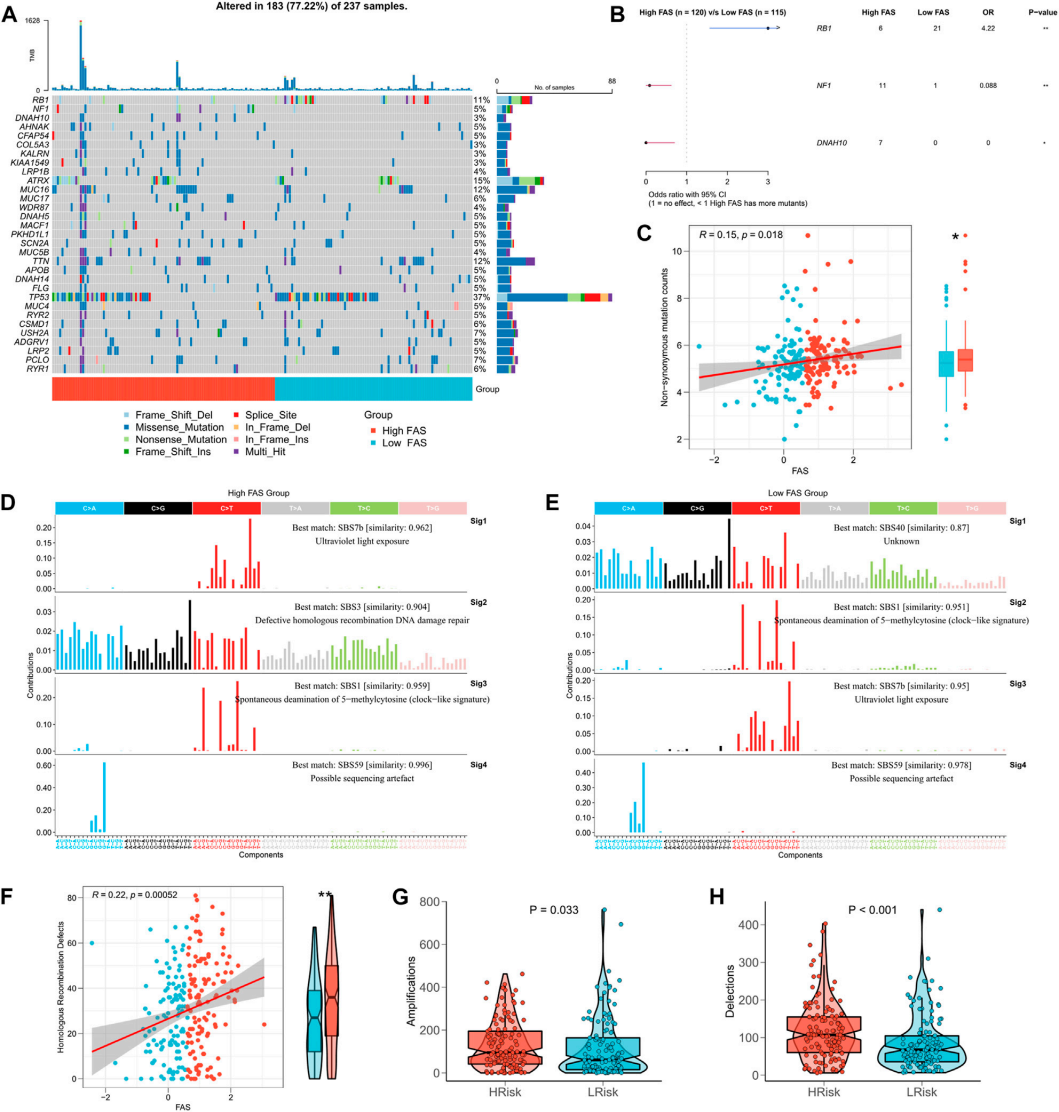
Association between FAS and tumor mutation burden. **(A)** TMB difference between the high- and low-FAS groups. **(B)** Genes with significant mutations among the high- and low-FAS groups (*p* < 0.05). **(C)** The relationship between FAS and non-synonymous mutation counts. **(D,E)** Homologous recombination repair in STS with different FAS. **(F)** The difference in homologous recombination defects between distinct FAS groups. **(G,H)** The copy number variation was significantly positively associated with FAS.

Based on the biological significance of TMB in tumors, we compared the difference in TMB between high- and low-FSA groups. The waterfall plot presents all the high-frequency mutation genes with the minimum number of mutations >8 (Figure 7A), where RB1, NF1, and DNAH10 mutation frequencies are the three genes with the most apparent mutation differences between high- and low-FAS groups. Figure 7B further demonstrated their mutation profiles, RB1 was most mutated in the low-FAS group, while NF1 and DNAH10 were more mutated in the high-FAS group. Similarly, we also found the FAS was positively correlated with the non-synonymous mutation counts (Figure 7C). Next, we found that the high FAS group was explicitly characterized by homologous recombination repair in terms of mutation. In particular, the STS patients in the high-FAS group had enhanced SBS3, implying a potential association between the mutation and homologous recombination defects (HRD) events (Figures 7D, E). The further analysis below confirmed the positive correlation between FAS and HRD (Figure 7F). Considering that HRD is one of the main factors of CNV amplification and deletion, we then analyzed the difference in CNV between the two FAS subgroups and discovered that the CNV was significantly increased in the high-FAS group (Figures 7G, H).

### The immunotherapy response prediction value of FAS

**FIGURE 8:**
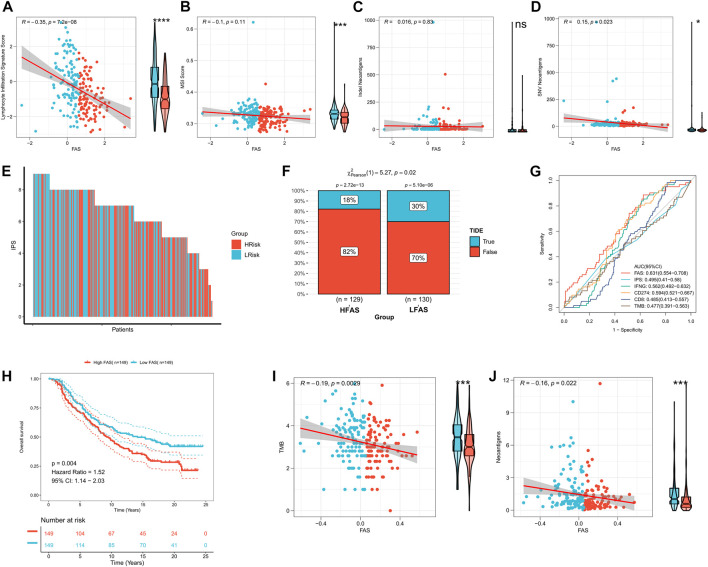
The impact of FAS on immunotherapy response in the STS cohort. **(A–D)** The association of FAS and lymphocyte infiltration signature score, MSI score, indel neoantigens, and SNV neoantigens. **(E)** The IPS of each STS individual. **(F)** The difference in TIDE between the two distinct FAS groups. **(G)** The AUC for immunotherapy response of FAS and other indexes. **(H)** Kaplan‒Meier curves of OS of two FAS groups in the Imvigor210 cohort treated with immunotherapy. **(I,J)** The correlation of FAS and TMB and neoantigens.

According to the above findings, the FAS was significantly related to the immune microenvironment. Thus, we explored whether the FAS can predict the immune response to STS. Initially, we compared the differences between STS cohorts with high and low FAS in terms of lymphocyte infiltration signature score, MSI score, Indel Neoantigens, and SNV Neoantigens. It was found that these scores (lymphocyte infiltration signature score, MSI score, and SNV Neoantigens) were highly expressed in the low FAS group, and there was a positive and negative correlation (Figures 8A–D). Subsequently, we further evaluated the differences in immunotherapy-related indicators in high and low FAS groups. The results revealed that the IPS of STS patients in the low FAS group was higher, suggesting that the patients in the low FAS were more effective for immunosuppressants at the immune checkpoints (Figure 8E; Supplementary Figure S3A). As shown in Figure 8F and Supplementary Figure S3B, the TIDE score also confirmed that more STS patients with low FAS responded effectively to Immunotherapy (TCGA cohort: low-FAS group (30%/70%) and high-FAS group (18%/82%); TARGET cohort: low-FAS group (50%/50%); high-FAS group (34%/66%)). These results indirectly suggest that the FAS may play a key role in predicting the effect of immunotherapy. Therefore, we further used the ROC curve to evaluate the predictive potential of the fatty acid-related score in predicting immune response. The results showed that the predictive potential of the fatty acid-related score was higher than other quantitative indicators (IPS, IFNG, CD274, CD8, and TMB) (Figure 8G; Supplementary Figure S3C). We further divided the patient’s receiving immunotherapy in the IMvigor210 dataset into the high FAS subtype and low FAS subtype. The survival analysis showed that the survival rate of STS individuals in the low FAS subtype was significantly ameliorated than that of patients with the higher FAS (Figure 8H). Finally, we also found that FAS scores were significantly negatively correlated with TMB and Neoantigens scores (Figures 8I, J). The above evidence showed that FAS could be a superior biomarker to predict the response to immunotherapy.

### *In vitro* validation of FRGs in STS

**FIGURE 9:**
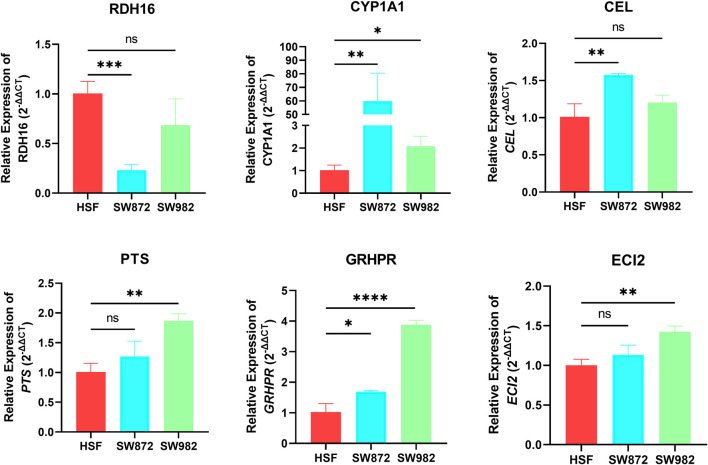
The mRNA expression of RDH16, CYP1A1, CEL, PTS, GRHPR, and ECI2 was examined by RT‒qPCR in HSF and STS cell lines.

To further verify the accuracy of our analysis results, we selected the top 8 characteristic FRGs with coefficients greater than 0 (Supplementary Table S5) to detect their expression in STS cells using RT-qPCR. As shown in Figure 9, we observe a significant abnormal expression in all FRGs. Among them, RDH16 was diminished in the SW872 compared to the HSF. On the contrary, the others selected signature FRGs exhibited an augmented trend in the STS cell. Especially for CYP1A1 and GRHPR, they were overexpressed both in SW982 and SW872 cell lines. Collectively, these results indirectly illustrate the importance of these signature FRGs in STS, which also confirms the reliability and accuracy of our previous bioinformatics analysis.

## Discussion

STS is a heterogeneous group whose current treatment strategies are increasingly adapted to a specific histological subtype, so it is urgent to find practical predictive factors for specific subtypes of STS. Recently, accumulating evidence has demonstrated that metabolic dysregulation is one of the hallmarks of tumor cells, leading to abnormal cellular biological behaviours of tumors such as proliferation, invasion, cell growth, and angiogenesis (Hanahan and Weinberg, 2011). In terms of energy metabolism of the tumor, fatty acid metabolism is a critical component that affects cancer cell biology, especially the synthesis of membrane-dependent lipid building blocks and energy production and storage (Koundouros and Poulogiannis, 2020). Additionally, it has been shown that excessive amounts of long-chain fatty acids trigger lipid-mediated toxicity or lipoapoptosis in cells (Schaffer, 2003). Furthermore, fatty acid metabolism has been explored for progress prediction and to characterize the effects of therapeutic and prognostic interventions for various cancers (Yamashita et al., 2017; Aladelokun et al., 2021). For instance, Feng Jiang et al. constructed a novel fatty acid predictive risk score model linked to the prognosis of glioma that could help with prognosis, prognosis, and immunotherapy response prediction (Jiang et al., 2022). Although numerous studies have focused on the predictive modelling of STS, few studies have investigated whether the model based on fatty acids could improve the clinical prediction of STS. Therefore, exploring the roles of distinct fatty acid metabolism characteristics in STS may contribute to a comprehensive understanding of the mechanism of fatty acid metabolism in the progression of STS, thus helping develop effective therapeutic strategies.

Here, we systematically investigated FRGs in STS for the first time. Initially, we identified 24 differentially expressed FRGs closely associated with the clinical survival of patients with STS through differential analysis and univariate Cox regression analysis. Among them, LTC4S exhibited a possible side as a protective factor for STS. Interestingly, previous studies have found that it is involved in cancers (Halvorsen et al., 2014; Wei et al., 2021), but its role in STS needs further investigation in the future. Subsequently, we performed the LASSO regression algorithm to further screen core fatty acid metabolism-related genes and constructed a FAS system based on the TCGA-STS cohort. The results reveal that FAS is an effective predictor of the prognosis prediction of STS patients. The performance of FAS was further confirmed by the external validation cohort from the TARGET and GEO databases. Concordantly, univariate, multivariate analysis, C-index, and ROC curves also revealed that FAS could be used as an independent prognostic indicator to predict the clinical survival rate of STS patients and outperformed other clinical factors. Additionally, we further constructed a nomogram that helped us better assess each STS patient’s clinical survival rate based on their clinical characteristics. Thus, we believe that the novel FAS can be applied to clinical decisions and trust that it will improve the prognosis of STS.

Tumor occurrence and development is an extremely complex process that involves multiple signaling pathway disorders (Chen et al., 2021). Consequently, we carried out functional enrichment analysis on the two FAS subgroups to explore the lurking biological mechanism. The GO and KEGG results implied that dysregulation of immunity and inflammation might play a crucial role in fatty acid metabolism on the STS’s tumorigenesis. In addition, GSEA further revealed that the fatty acid metabolism related to some cancer-related pathways and immunity that have been proven implicated in STS. For instance, Gang Tan et al. demonstrated that PLA2G10 promotes the expression of cyclin E1 and CDK2, resulting in the cell-cycle progression of soft tissue leiomyosarcoma (Tan et al., 2020). Similarly, our results also revealed that the FAS was positively relevant to WNT beta-Catenin and TGF beta signalling. A previous study proved that activating the TGF-β1/Smad signaling pathway *via* SHCBP1 could facilitate metastasis and result in a poor prognosis of synovial sarcoma (Peng et al., 2017). WNT beta-Catenin is widely known in the progress of epithelial origin neoplasm and could serve as a potential therapeutic opportunity for STS (Barraud-Hadidane et al., 1987). As we all know, the p53 tumor suppressor pathway is a vital signalling pathway in the prevention of tumor formation, and the FAS was negatively related to the p53 pathway, which may be one of the causes of the STS in the low FAS group had a better prognosis (Muller and Vousden, 2014). Thus, the result and research hinted that fatty acid metabolism might regulate these pathways to affect the tumorigenesis, progression, and prognosis of STS, but the specific association needs further elucidation in the future.

It has been well demonstrated that the tumor immunity microenvironment is a complex and dynamic ecosystem consisting of the tumor, immune, and stromal cells (Zuo et al., 2020). Notably, our results reveal that the tumor immunity microenvironment differed between the distinct FAS group patients. The immune checkpoint expression and immunity score were markedly strengthened for the STS cohort in the low-FAS group. This preliminary hinted that immune status between STS patients in high and low FAS groups might be relevant to clinical outcomes, although the detailed association between TMB and prognosis remains controversial and varies across cancer types (Wang et al., 2021; Qing et al., 2022). In addition, we investigate the distinct composition of various immune cell types in low and high FAS groups in more detail. Consequently, several types of immune cells were notably strengthened in low-FAS patients, such as M1 macrophages and CD8 T-cell. Extensive research focusing on the tumor microenvironment has demonstrated the immune cell pivotal role in cancer dissemination and progression (Zhang et al., 2020). It has well know that the M1 macrophages were closely relevant to inflammatory immune response, exhibiting a principal role in the anticancer effect (Komura et al., 2015). For instance, a previous study has confirmed that the transformation from the M2 macrophage phenotype to an M1 macrophage phenotype induced by pAbM could inhibit tumor progression (Liu et al., 2015). Traditionally, CD8^+^ cells have been considered the primary mediators of T cell antitumor responses, and the high infiltration of CD8^+^ T-cells was a superior prognosis predictor (Zuo et al., 2020). Mengzhou Guo et al. demonstrated that CD8^+^ T-cells represent a favourable prognostic biomarker for hepatocellular carcinoma, which is consistent with our result (Guo et al., 2020). Hence, these results revealed that the low-FAS group patient might be companies with an enhanced immunity status, which may account for an improved prognosis.

In many cases, tumor metabolism is related to immunotherapy. Previous studies have revealed that fatty acid metabolism could influence the immunotherapeutic effect for cancer individuals (He et al., 2021). Considering the association of FAS with the immune microenvironment, we further evaluated the ability of FAS to predict immunotherapeutic response to help us distinguish candidates applied for immune therapy in clinical practice. In addition, our results indicated that the patients in the low-FAS group have a higher IPS score, suggesting they have better efficacy for immune checkpoint inhibitors, which is similar to previous studies (Zhu et al., 2022). Additionally, we conducted a K-M survival analysis using a validation cohort and found that the patients receiving immunotherapy with lower FAS had an improved prognosis. TMB, as an emerging biomarker, has received increasing attention in tumor immunotherapy (Valero et al., 2021). A high TMB has been identified as relevant to the better efficacy of immune checkpoint inhibitor therapy in tumors (Galuppini et al., 2019). Consistently, we demonstrated that there was a negative correlation between FAS and TMB scores, implying that STS patients with lower FAS are candidates individual for immunotherapy. Collectively, it is reasonable to believe that the FAS are better predictors of immunotherapy, which may help to individualize immunotherapy and further improve treatment outcomes for STS.

Despite these compelling and interesting findings, several limitations to the current study remain. Initially, since some important clinical features were not available in a public database, such as tumor staging, which may limit us in predicting the prognosis for STS patients more accurately. Secondly, our current results indicated that the FAS system might not be ideal in predicting progression-free survival for STS, which may be due to the sample limitation of the TCGA-STS cohort and the high heterogeneity of the STS itself. Last but not least, although our study verified our model’s reliability through internal, external, and *in vitro* experiments validation, future studies focuing on the clinical application of the novel FAS are also necessary. Therefore, it will be important to collect more clinical STS samples in the future to verify our results further to achieve an accurate diagnosis and treatment of STS better.

## Conclusion

In summary, this study is the first to conduct a comprehensive and systematic analysis of fatty acid metabolism-related genes in STS and successfully establish a fatty acid-related risk score. The scoring systems based on fatty acid metabolism can be used for the prognosis and immunotherapy evaluation of STS patients, thereby facilitating and optimizing the personalized treatment of STS patients. From a long-term perspective, this study identified the crucial preclinical significance of fatty acid metabolism for STS research and brought a novel idea and strategies for STS individualized treatment.

## Data Availability

The original contributions presented in the study are included in the article/Supplementary Material, further inquiries can be directed to the corresponding authors.

## Abbreviations

DEG, differential gene expression; FASN, fatty acid synthase; FAS, fatty acid metabolism score; FNCLCC, french federation nationale des centres de lutte contre le cancer; FRGs, fatty acid metabolism-related genes; GEO, gene expression omnibus; GO, gene ontology; GSEA, gene set enrichment; HRD, homologous recombination defects; IPS, immunophenoscore; KEGG, kyoto encyclopedia of genes and genomes; LASSO, least absolute shrinkage and selection operator; PFS, progression-free survival; RT-qPCR, real-time quantitative polymerase chain reaction; ssGSEA, single sample gene set enrichment analysis; STS, soft tissue sarcoma; TARGET, therapeutically applicable research to generate effective treatments; TCGA, the cancer genome atlas; TIDE, tumor immune dysfunction and exclusion; UCSC, university of california santa cruz.
