# Supplementary figures and images for: Integration analysis based on fatty acid metabolism robustly predicts prognosis, dissecting immunity microenvironment and aiding immunotherapy for soft tissue sarcoma
Source: Front Genet. 2023 Mar 30;14:1161791. doi: 10.3389/fgene.2023.1161791 (PMC10097927; doi:10.3389/fgene.2023.1161791)

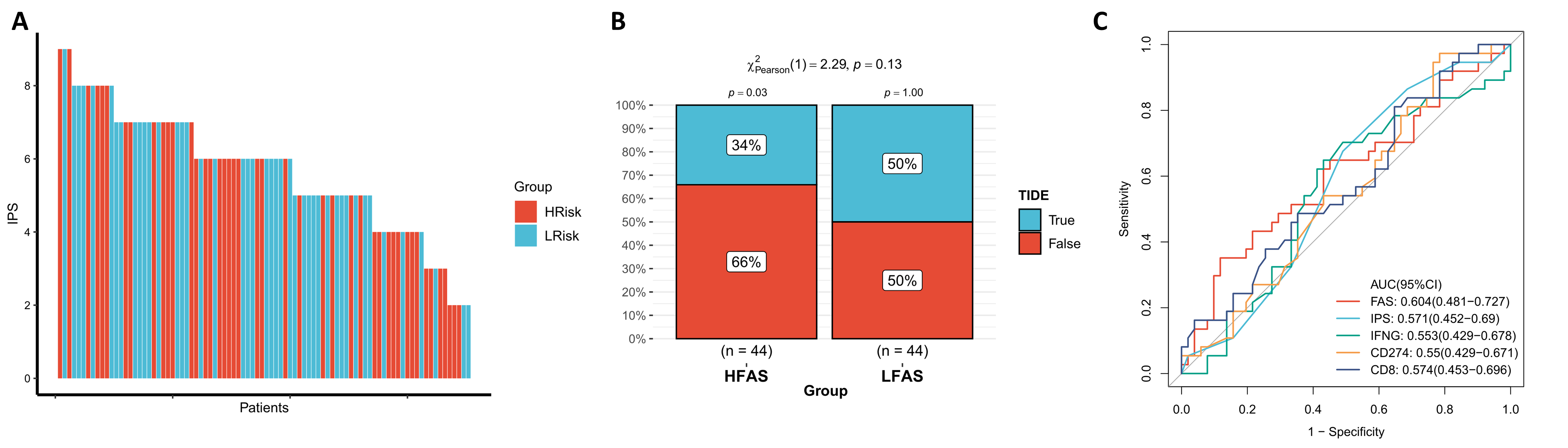

Supplement: Supplementary file 3 [file Image3.TIF]

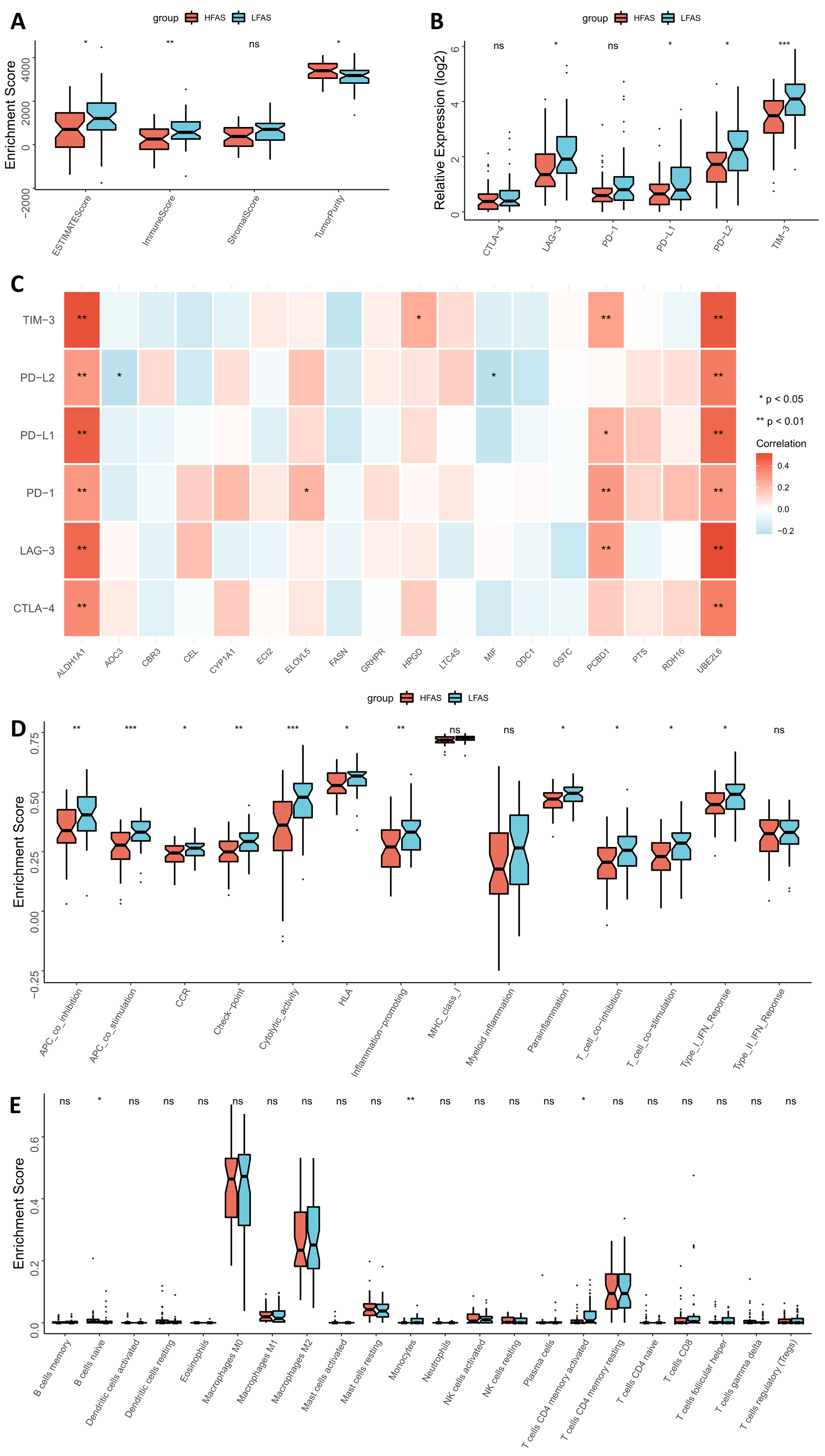

Supplement: Supplementary file 4 [file Image2.TIF]

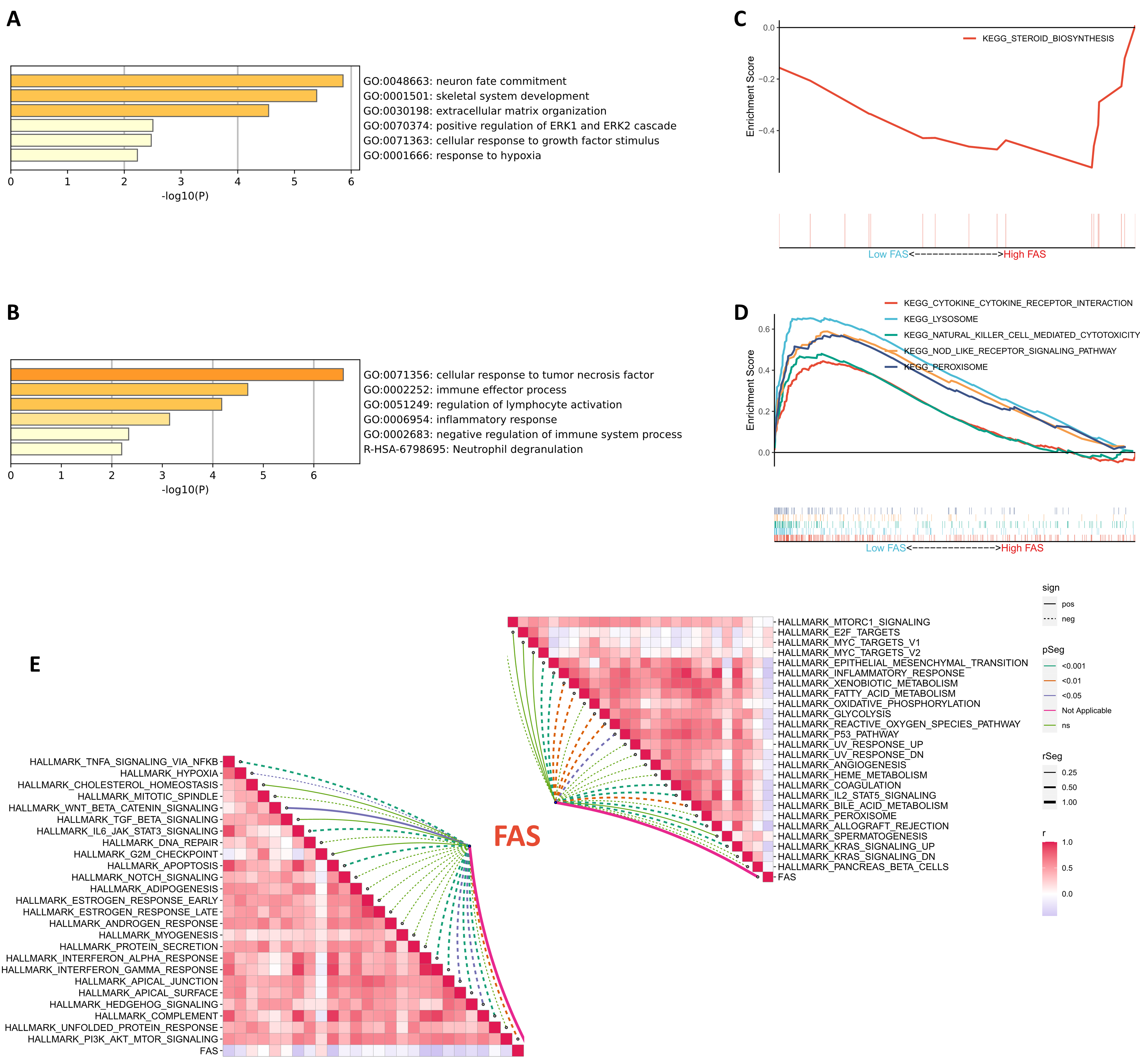

Supplement: Supplementary file 5 [file Image1.TIF]
